# Hydrogen sulfide mitigates skeletal muscle mitophagy‐led tissue remodeling via epigenetic regulation of the gene writer and eraser function

**DOI:** 10.14814/phy2.15422

**Published:** 2022-08-19

**Authors:** Mahavir Singh, Sathnur Pushpakumar, Yuting Zheng, Rubens P. Homme, Irina Smolenkova, Sri Prakash L. Mokshagundam, Suresh C. Tyagi

**Affiliations:** ^1^ Department of Physiology University of Louisville School of Medicine Louisville Kentucky USA; ^2^ Division of Endocrinology, Metabolism and Diabetes and Robley Rex VA Medical Center University of Louisville School of Medicine Louisville Kentucky USA

**Keywords:** 1‐carbon metabolism, cystathionine β synthase, homocysteine, mitochondria

## Abstract

Ketone bodies (KB) serve as the food for mitochondrial biogenetics. Interestingly, probiotics are known to promote KB formation in the gut (especially those that belong to the *Lactobacillus* genus). Furthermore, *Lactobacillus* helps produce folate that lowers the levels of homocysteine (Hcy); a hallmark non‐proteinogenic amino acid that defines the importance of epigenetics, and its landscape. In this study, we decided to test whether hydrogen sulfide (H_2_S), another Hcy lowering agent regulates the epigenetic gene writer DNA methyltransferase (DNMT), eraser FTO and TET2, and thus mitigates the skeletal muscle remodeling. We treated hyperhomocysteinemic (HHcy, cystathionine beta‐synthase heterozygote knockout; CBS^+/−^) mice with NaHS (the H_2_S donor). The results suggested multi‐organ damage by HHcy in the CBS^+/−^mouse strain compared with WT control mice (CBS^+/+^). H_2_S treatment abrogated most of the HHcy‐induced damage. The levels of gene writer (DNMT2) and H3K9 (methylation) were higher in the CBS^+/−^ mice, and the H_2_S treatment normalized their levels. More importantly, the levels of eraser FTO, TET, and associated GADD45, and MMP‐13 were decreased in the CBS^+/−^ mice; however, H_2_S treatment mitigated their respective decrease. These events were associated with mitochondrial fission, i.e., an increase in DRP1, and mitophagy. Although the MMP‐2 level was lower in CBS^+/−^ compared to WT but H_2_S could further lower it in the CBS^+/−^ mice. The MMPs levels were associated with an increase in interstitial fibrosis in the CBS^+/−^ skeletal muscle. Due to fibrosis, the femoral artery blood flow was reduced in the CBS^+/−^ mice, and that was normalized by H_2_S. The bone and muscle strengths were found to be decreased in the CBS^+/−^ mice but the H_2_S treatment normalized skeletal muscle strength in the CBS^+/−^ mice. Our findings suggest that H_2_S mitigates the mitophagy‐led skeletal muscle remodeling via epigenetic regulation of the gene writer and eraser function.

## Graphical abstract

Abnormal mitophagy in the CBS^−/+^ mice skeletal muscle is mitigated by the H_2_S treatment successfully.

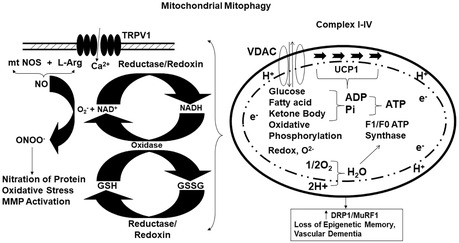

## 1 INTRODUCTION

Elevated levels of homocysteine (Hcy, i.e., hyperhomocysteinemia, HHcy) are associated with frailty in elders, skeletal muscle malfunctioning, metabolic injury, and associated mortality (Hoogeveen et al., 2000; Kado et al., 2002; Veeranki & Tyagi, 2013; Wong et al., 2013). HHcy patients also exhibit below normal body weights and musculoskeletal abnormalities (Kalra et al., 1985). It is known that the homozygous cystathionine β‐synthase (CBS) knockout mice (CBS^−/−^) die shortly after birth with multiple musculo‐skeletal defects. In addition, cystathionine gamma‐lyase (CSE) knockout mice exhibit lethal myopathies (Ishii et al., 2010; Watanabe et al., 1995). However, it remains enigmatic how HHcy impairs skeletal muscle biology and its function. Although, nutritional supplementation and control of HHcy are very attractive strategies but so far have yielded ambivalent outcomes, and subsequent results (Beard Jr. & Bearden, 2011; Maron & Loscalzo, 2009). In the past, it was proposed that Hcy reduction would accompany by hydrogen sulfide (H_2_S) production enhancement that could be beneficial rather than simply lowering the plasma Hcy levels (Beard Jr. & Bearden, 2011). However, no such studies were planned or conducted along these lines. In fact, lowering the Hcy alone showed very little success indeed (Beard Jr. & Bearden, 2011). Thus, our strategy of employing the CBS^+/−^ heterozygous knockout mice in this work not only eliminates Hcy from the circulation but also produces H_2_S. In previous studies, transgenic expression of the CBS gene in mice was carried out to reduce Hcy plasma levels and prevent HHcy phenotypes, however, it led to alterations in other amino acid content in the liver (Wang et al., 2004). However, overexpression of the CBS gene was reported to cause a positive neuronal phenotype in the transgenic mice (Bayraktar & Kreutz, 2018a; Butler et al., 2006; Jarome et al., 2018; Régnier et al., 2012). In another study, transgenic mice expressing low levels of human CBS gene after inactivation of mouse gene were reported as a model for investigating HHcy with altered blood coagulation phenotype (Maclean et al., 2010).

As we know that significance and mechanistic aspects of H_2_S signaling in skeletal muscle physiology and function are not completely understood especially regarding the adaptive responses. This study has been designed to examine the molecular changes, and functional consequences of the transient but systemic increase in H_2_S production versus prolonged H_2_S production in three key determinants of skeletal muscle adaptability encompassing the vasculogenic, atrophy, and regeneration attributes. It is expected that the positive results of this study will have direct translational potential regarding H_2_S as a potential therapeutic agent for musculo‐skeletal, and connective tissue disorders. Furthermore, researchers have noted a progressive reduction in the plasma thiol levels with increasing age despite increased plasma cysteine contents that are correlated with diminished physical performance in elderly people (Droge, 2005). It is well established that aging enhances plasma Hcy levels, and Hcy competes with cysteine transporters for the cysteine, as it appears that there might be a greater need for a greater cysteine supply within the skeletal muscle tissue (Ishii et al., 2010; Veeranki & Tyagi, 2013). In addition, the short life span of the H_2_S molecule per se (Henderson et al., 2011) limits the use of chemical H_2_S donors. Thus, NaHS administration would help supplement the H_2_S presence in locally defined regions of skeletal muscle where it is most necessary. We opine that the CBS transgene would cater to those needs.

Our proposal would not solely unravel the relative contribution(s) of the presence of H_2_S or lack of HHcy, it will also ensure the presence of H_2_S (in CBS^+/−^ mice) during tissue perfusion, and muscle atrophy. In addition, the proposed study has the potential to identify key signaling pathways that invariably govern the skeletal muscle adaptability that is most likely affected by the HHcy condition and the therapeutic H_2_S production. A positive outcome from the study will provide mechanistic aspects of the adaptive response after ischemia, and related injury. Furthermore, it may also provide a newer mechanism(s) of skeletal muscle metabolism regarding insulin resistance, mitochondrial dynamics, antioxidant capability, and tissue remodeling. The results from this study will help aid in formulating preventive or curative strategies and assess the usefulness of H_2_S treatment in numerous forms of muscle myopathy.

In this work, we compared the influence of HHcy versus persistent H_2_S signaling in skeletal muscle perfusion, regeneration, and muscle frailty. Our strategy of employing the CBS heterozygous knockout mice would also help cater to the needs of HHcy patients with genetic mutation either in the methylenetetrahydrofolate reductase (MTHFR), CBS, or the methionine synthase (MS) gene. The MTHFR converts 5,10‐methylenetetrahydrofolate to 5‐methylenetetrahydrofolate while 5‐methylenetetrahydrofolate donates a methyl group for the conversion of Hcy to methionine the second reaction is catalyzed by the MS whose activity depends on the presence of vitamin B_12_ (folate; Al‐Batayneh et al., 2018; Goyette et al., 1995), thus the absence of folate also causes HHcy. Recent studies showed that lactic acid‐producing bacteria such as Lactobacillus can produce folate (Albano et al., 2020), thus the Lactobacillus‐centered probiotics are capable of preventing the HHcy. The presence of CBS expression in our model would effectively remove the circulating plasma Hcy levels. Given that mouse skeletal muscles lack key enzymes involved in H_2_S production, the mice expressing the CBS gene would serve as a model for understanding the detoxification of Hcy, and the biology of H_2_S signaling in many different disease contexts relevant to muscle biology, and aging.

## 2 MATERIALS AND METHODS

### 2.1 Administration of NaHS

Wildtype (C57BL/6J) and a breeding pair of the CBS^+/−^ mice were obtained from the Jackson Laboratory. Mice were divided into four experimental groups; each group consisted of 5–12 males and females between 10 and 12 weeks of age. The body weights of the mice were measured. The H_2_S (sodium hydrosulfide hydrate; Sigma–Aldrich, Cas # 207683–19‐0; dose: 10 mg/kg was dissolved in drinking water to create a concentration of 0.05 mg/ml) and given to mice for 6 weeks. Each mouse drinks approximately 5 ml/day. A total of 25 ml was prepared fresh each day for each mouse (Kamat et al., 2013; Majumder et al., 2018). A minimum of 5–12 mice/group were used during the experiments.

### 2.2 Blood pressure

Blood pressure was measured via tail vein through non‐invasive Coda instruments (Kent Scientific; Bisserier et al., 2021; George et al., 2020).

### 2.3 Creatinine kinase assay

Multi‐organ specific injury was determined by measuring creatine kinase (CK) activity as described earlier (Miller et al., 2000). A modified version of CK assay is available wherein either plasma or serum samples could be mixed with 1 μl of activator and loaded onto the pre‐made CK gel as instructed by the manufacturer (QuickGel® CK Vis isoenzyme procedure; Helena Laboratories). The gels are typically run at 400 V for 4:15 min. The standard (ST) amounts of CK isoforms (that are supplied by the kit manufacturer as loading controls) were also loaded in parallel to the samples for comparison (Singh et al., 2020; Stanisic et al., 2021).

### 2.4 Western blotting

All reagents and chemicals were purchased from Sigma‐Aldrich or available elsewhere but with the highest grade. The antibodies for ten‐eleven translocate 2 (TET2, #36449), fat obese associated protein (FTO, #31687), di‐methyl‐histone (Lys9) (H3K9, #4658), and matrix metalloproteinases‐13 (MMP13, #69926) were ordered from Cell Signaling Technology. Antibodies for dynamin‐related protein‐1 (DRP1, ab184247), muscle RING‐finger protein‐1 (MuRF1, ab201941) were purchased from Abcam. Whereas DNA 5‐cytosine methyltransferase 2 (DNMT2, sc‐365,001), musclin (sc‐365,631), growth arrest, and DNA damage‐45 protein (GADD45B, sc‐377,311), glyceraldehyde 3‐phosphate dehydrogenase (GAPDH, sc‐137,179) were purchased from Santa Cruz Biotechnology. Briefly, after sacrifice, the gastrocnemius muscle was removed, and stored at −80°C. Protein samples were extracted by homogenization in an ice‐cold RIPA buffer containing protease inhibitor cocktail. The homogenates were centrifuged at 13,100 *g* for 20 min at 4°C. The supernatants were collected. Protein concentration was measured with Bradford Assay. Equal amounts of protein samples (100 mg) were resolved on SDS‐PAGE (10%) and then transferred to PVDF membranes. Immunoblotting with primary and secondary antibodies was performed according to the manufacturers' protocol. ECL Immobilon Forte Western HRP substrate (Millipore) was used to image the blots in a Bio‐Rad Chemi Doc System. The intensities of the bands were normalized via GAPDH for all the target proteins. The quantification was performed using Image Lab Software (Bio‐Rad; Majumder et al., 2018).

### 2.5 Zymography

Using Zymography the proteolytic activity of MMP‐2 on the substrate gel was quantitated. For the preparation and running of the gel, the separation of the band was carried out on 10% of Acrylamide: bis‐Acrylamide (29:1) in 0.375 M Tris–HCl, pH 8.80, 0.1% SDS, and 3 mg/ml of gelatin. Stacking gel had 4% of acrylamide‐bis acrylamide, and 0.1% SDS in 0.125 M Tris–HCl pH 6.80. Sample loading buffer had 50 mM Tris–HCl, pH 6.8, 10% glycerol, 1% SDS, 0.01% bromophenol blue. Collagenase buffer was comprised of 50 mM Tris–HCl pH 7.6, 0.2 M NaCl, 5 mM CaCl2, and 0.001% Brij‐35. The staining solution contained 0.1% Coomassie brilliant blue R250, 40% methanol, and 10% acetic acid. The distaining solution had 40% methanol and 10% acetic acid. The loaded sample consisted of serum (1 μl) with sample loading buffer on the SDS‐PAGE gel. The gel was run at 120v until the dye ran off the gel. The gel was then soaked in 2.5% Triton x‐100 on a shaker for 2 h, followed by washes (four times) in the distilled water. Then soaked in collagenase buffer and incubated at 37°C overnight on a shaker. Prior to staining, the gel was briefly rinsed in the distilled water and then stained with a staining solution for 2 h. The gel was then de‐stained with a distaining solution for 3 h. A bio‐Rad system was used to scan the gel, and the band was quantitated (Tyagi et al., 2011).

### 2.6 Histological analysis

Collagen fibrosis was estimated by labeling with the Masson trichrome‐blue stain of the histological sections, as previously described(George et al., 2020; Majumder et al., 2018; Singh et al., 2021).

### 2.7 Measurement of tissue perfusion and red blood cells (RBC) density

Tissue perfusion rates were performed daily via Full‐Field Laser Perfusion Imager (Moor Instruments). The mice were then euthanized; soleus and gastrocnemius muscle tissues were harvested from CBS^+/−^ and WT mice treated with or without H_2_S. Free tissue sulfide levels were assayed in two different muscles (tibialis anterior and gastrocnemius) at the end of the treatment using methods reported before(Rivers et al., 2012). Plasma levels of Hcy were also determined by the HPLC‐UV method (Amarnath et al., 2003). We also assessed the changes in the angiogenic program by protein arrays from all the groups to identify the vasculogenic changes. The red blood cell (RBCs) density was also measured to know the effects of H_2_S treatment on the blood flow in the limb of CBS^+/−^ mice. As RBCs contain a high concentration of hemoglobin (Hb), which binds reversibly with O_2_ thus the amount of O_2_ released in the peripheral tissue, including the muscle depends on the quantity of the circulating RBCs (Cabrales et al., 2008).

### 2.8 Grip test

Grip strength of the forelimbs (front 2 paws), and all limbs (4 paws) was evaluated using the Grip Strength Meter (Bioseb, BIO‐GS3). As per the manufacturer's protocol, mice were held by the tail, lowered toward the apparatus, and allowed to grab the metal grid using 2 or 4 paws. The mice were pulled backward™ horizontally, and the force applied to the grid just before they lost their grip was recorded as the peak tension (converted to grams by the transducer). Peak force was measured two times in succession for each mouse for the front 2 paws, and all 4 paws. The mean value of both trials was used for analysis. Mice were given a minimum break of 5 min between trials (Majumder et al., 2018).

### 2.9 Statistical analysis

Results were expressed in mean ± SE. For tissue and molecular analysis, a minimum of 5–12 mice per group were used. For comparison of the parameters between two groups, Student *t* test, and one‐way ANOVA was used to derive the significance of group means. Statistical parameters were calculated using primer software. The acceptable level of significance was *p* < 0.05. Russ Lenth's power and sample size software were used to calculate the minimum number of animals required per each group.

## 3 RESULTS

**FIGURE 1:**
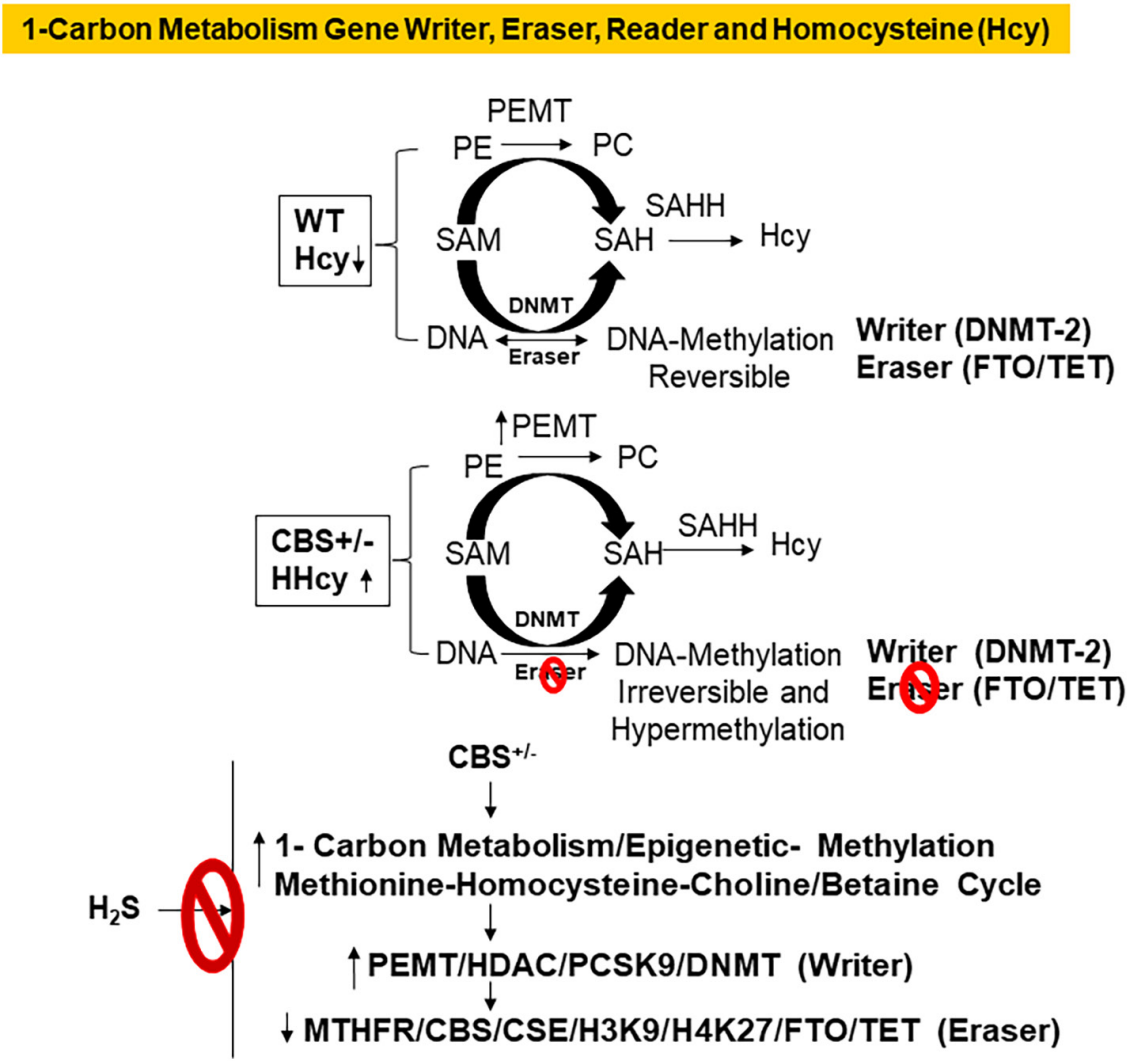
We hypothesize that “1‐carbon metabolism” controls epigenetic information by gene writer, and eraser, and its dysregulation leads to hyperhomocysteinemia (HHcy) in the CBS^+/−^mice. The H_2_S treatment helps mitigate this dysfunction.

**FIGURE 2:**
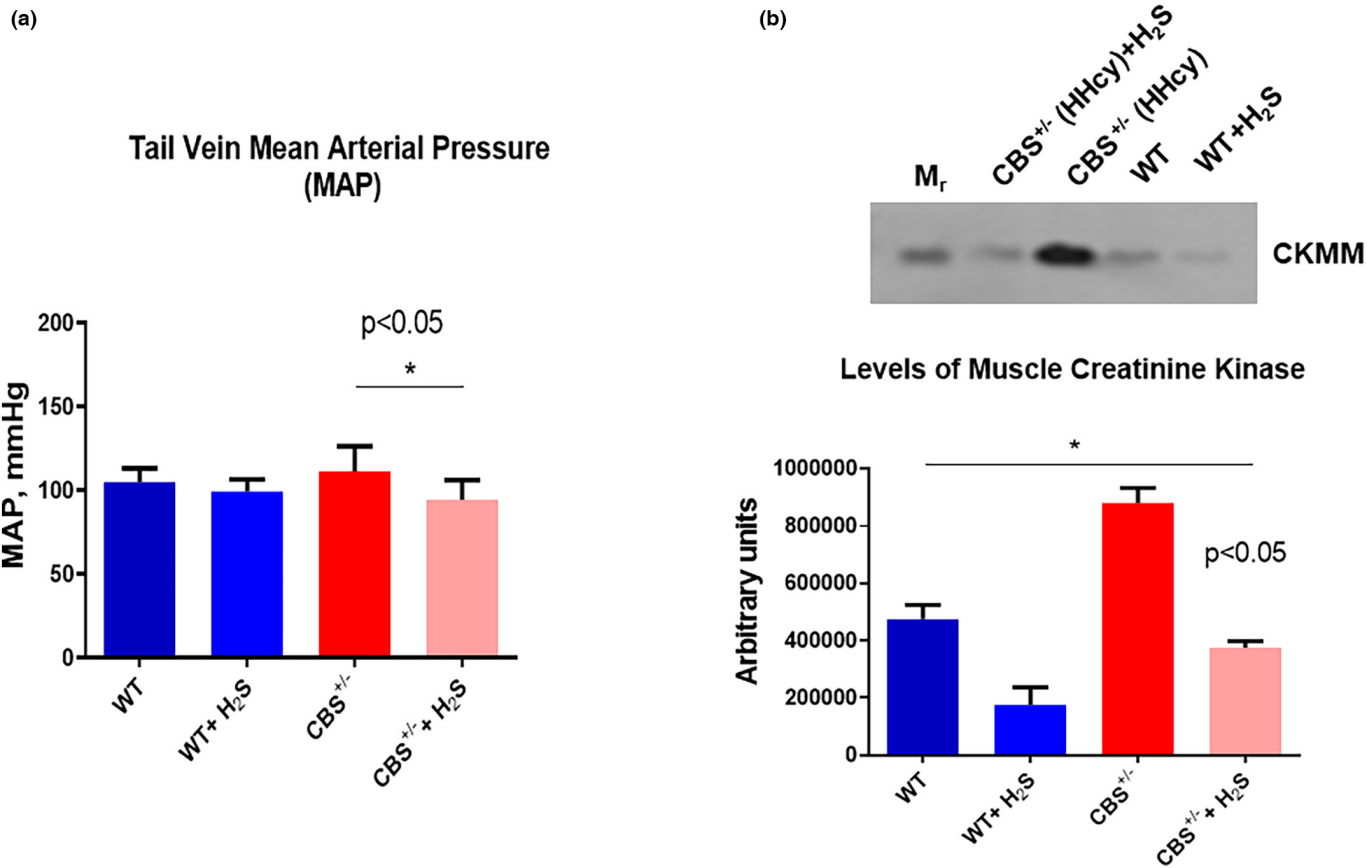
CBS^+/−^mice are hypertensive due to hyperhomocysteinemia (HHcy). Thus, the tail vein mean arterial pressure (MAP) is higher in these CBS^+/−^ mice as compared to the WT (CBS^+/+^) mice (a). The H_2_S treatment normalizes the MAP in the CBS^+/−^ mice. The levels of creatinine kinase (CK; the injury marker), in the muscle (CKMM) were found to be significantly higher (*p* < 0.01; *n* = 3) in the CBS^+/−^mice as compared to the WT controls (b). The H_2_S treatment mitigated the CK levels in the CBS^+/−^mice. The bar graph shows the respective quantitative values of the CK (b).

Epigenetic gene regulation by gene writer and eraser control the genotype, and phenotype of a cell (Gu et al., 2020). Homocysteine (Hcy) production is the hallmark of epigenetic gene methylation (Gavin et al., 2013). Thus, we tested the hypothesis that hydrogen sulfide (H_2_S); a gaseous Hcy lowering agent, mitigates skeletal mitophagy led remodeling by epigenetic regulation of the gene writer, and eraser function during hyperhomocysteinemia (HHcy) (Figure 1). Hcy induces systemic hypertension and causes multi‐organ systemic damage. We measured tail‐vein mean arterial pressure (MAP), and the creatinine kinase (CKMM), the skeletal muscle isoform, a marker of injury. The results depicted higher MAP in CBS^+/−^ and robust injury to organs during HHcy (Figure 2). H_2_S treatment mitigated MAP, and the multi‐organ damage in CBS^+/−^successfully. MAP is a critical hemodynamic factor, and thus an absence of proper regulation of MAP can have important pathophysiological consequences on organ function, including the skeletal muscle. In fact, all 3 gaseous transmitters (NO, CO, and H_2_S) have vasoactive effects on the body, and hence it is likely that they all participate in blood pressure regulation (Cacanyiova et al., 2016).

**FIGURE 3:**
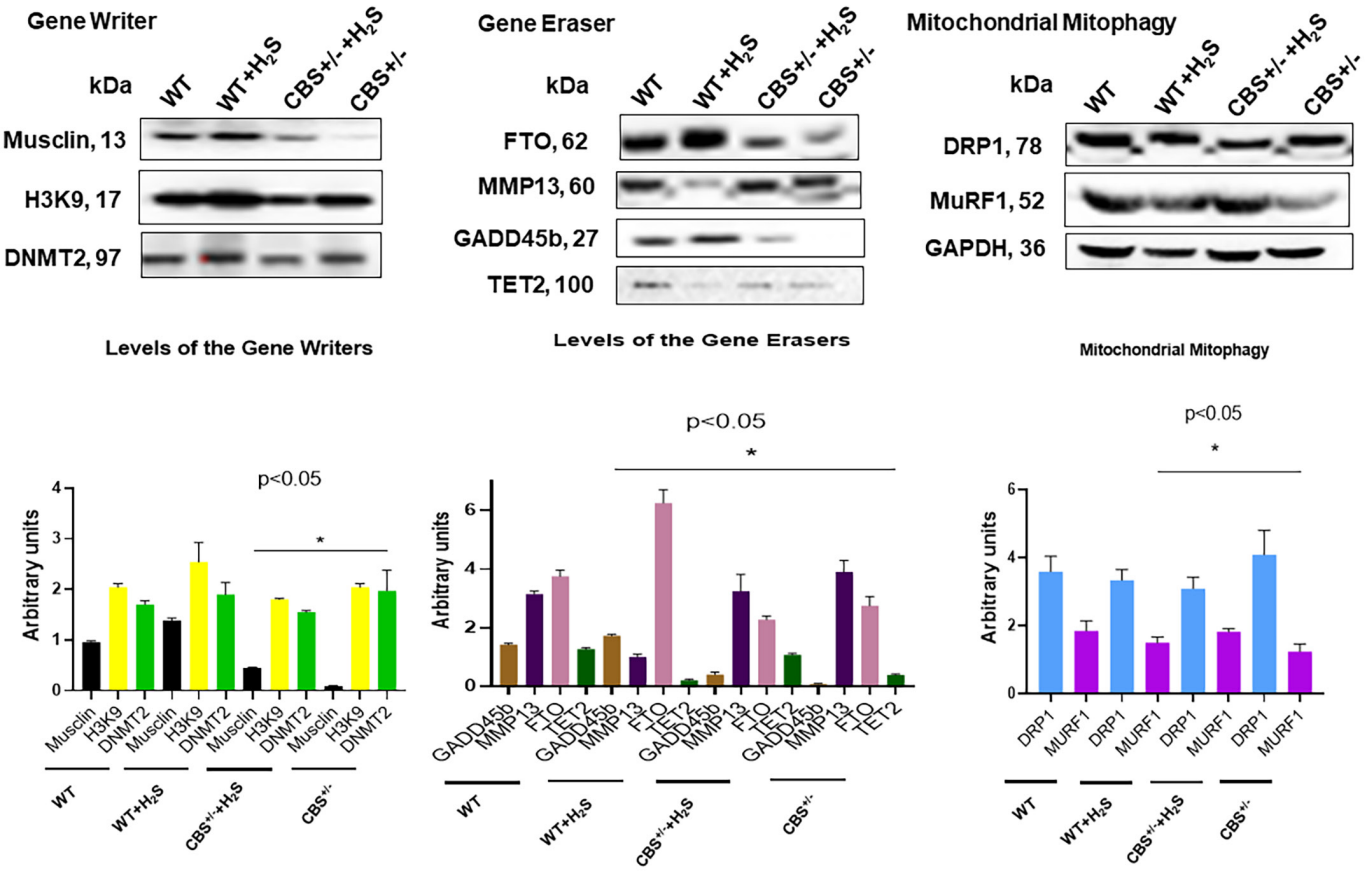
The levels of gene writer (DNMT2), skeletal muscle musclin, and the histone 3 lysine 9 methylation (H3K9) were measured. The levels of gene eraser, fat obese associated protein (FTO), ten‐eleven translocate (TET2), growth arrest and DNA damage‐45 protein (GADD45b), associated matrix metalloproteinase‐13 (MMP13) were determined. The levels of mitochondrial mitophagy were also determined by calculating the ratios of the fission protein (DRP1), and fusion protein (MuRF1), *p < 0.05; *n* = 5–12.

Musclin is a myokine that is produced by skeletal muscle. Its functions are to protect against various forms of injuries. Skeletal muscle secreted musclin is released into the systemic circulation wherein it increases the mitochondrial oxidative capacity during exercise in an order to increase physical endurance. Muscle physiological output is activated via the Akt phosphorylation and that seems to inhibit the forkhead box 01 transcription factor which in turn leads to the release of musclin‐gene encoded protein (Subbotina et al., 2015). Thus, we measured the musclin levels in our mice groups and that was found to be significantly abated in the CBS^+/−^strain. The H_2_S treatment increased the musclin levels in the CBS^+/−^ mice. To determine the levels of epigenetic gene writer, eraser, and mitochondrial remodeling aspects, we then measured the DNA 5‐cytosine methyltransferase 2 (DNMT2), di‐methyl‐histone (Lys9) (H3K9, methylation), fat obese associated protein (FTO), ten‐eleven translocate 2 (TET2), growth arrest and DNA damage‐45 protein (GADD45b), matrix metalloproteinases‐13 (MMP‐13), dynamin‐related protein‐1 (DRP1), and the muscle RING‐finger protein‐1 (MuRF1) respectively (Bayraktar & Kreutz, 2018b; Gu et al., 2020; Ijiri et al., 2005; Mathiyalagan et al., 2019; Sendžikaitė et al., 2019). The levels of writers were increased, and erasers decreased during mitophagy. However, the H_2_S treatment mitigated these changes (Figure 3).

**FIGURE 4:**
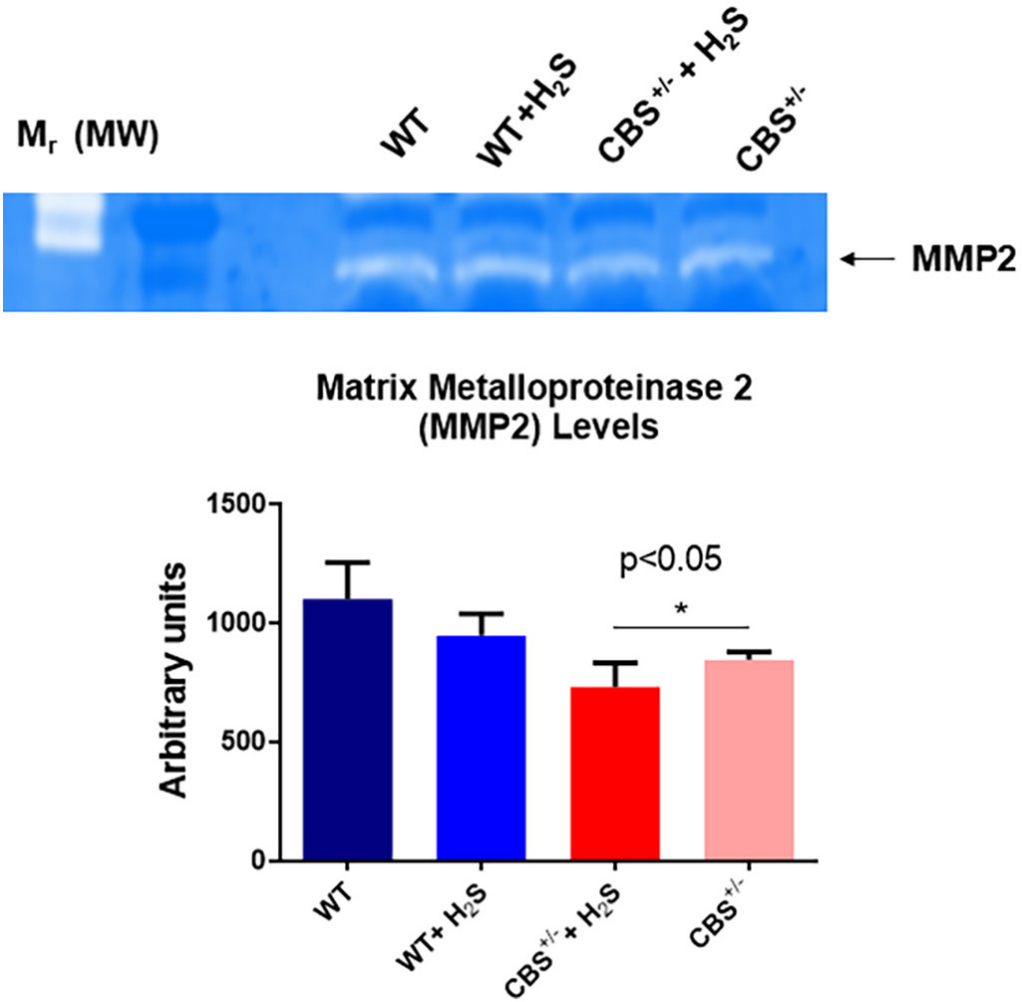
The skeletal muscle remodeling was measured by in‐gel zymography for matrix metalloproteinase‐2 (MMP2). The levels of MMP2 were mitigated by the H_2_S treatment. The lower levels of MMP2 activity in CBS^+/−^ may be due to inhibition of MMP‐zymographic‐activity by sulfur‐containing agents which may not be the case in the WT controls, *p < 0.05; *n* = 5–12.

**FIGURE 5:**
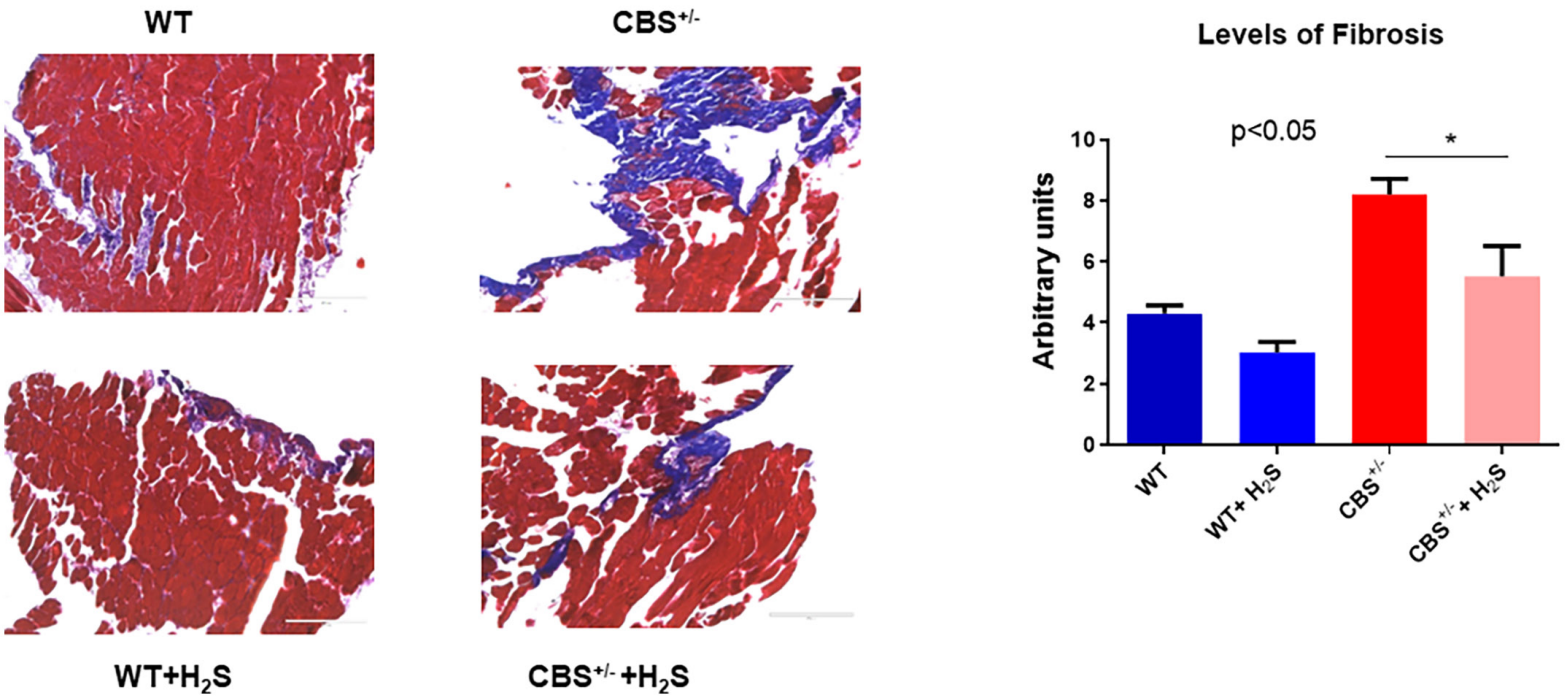
The skeletal muscle fibrosis was measured by trichome‐blue Masson staining methods. The results suggest robust interstitial fibrosis in the CBS^+/−^ mice muscle. The H_2_S treatment mitigated this fibrosis in the muscle, *p < 0.05; *n* = 5–12.

**FIGURE 6:**
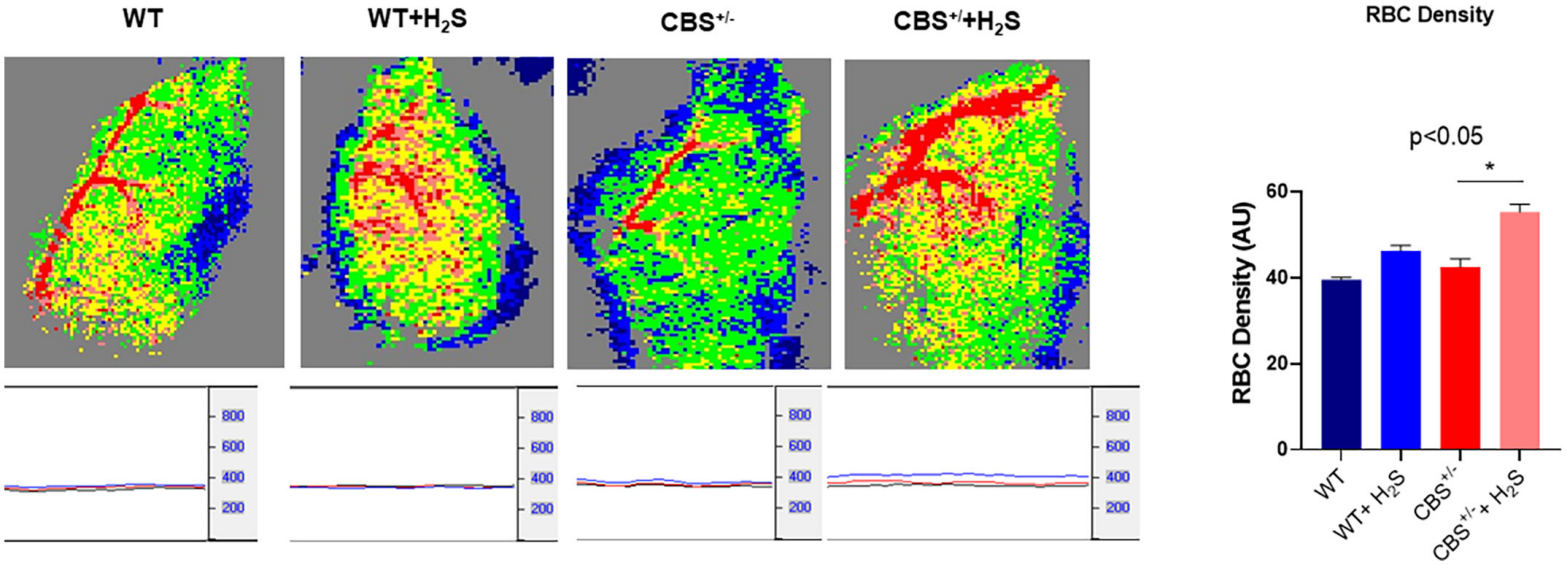
To determine whether fibrosis and skeletal muscle remodeling compromise limb blood flow, we measured the perfusion via a full‐field laser perfusion imager procedure. The flow was decreased in the CBS^+/−^ mice as compared to the WT controls. H_2_S treatment normalized the blood flow in the limb of CBS^+/−^ mice, *p < 0.05; *n* = 5–12.

**FIGURE 7:**
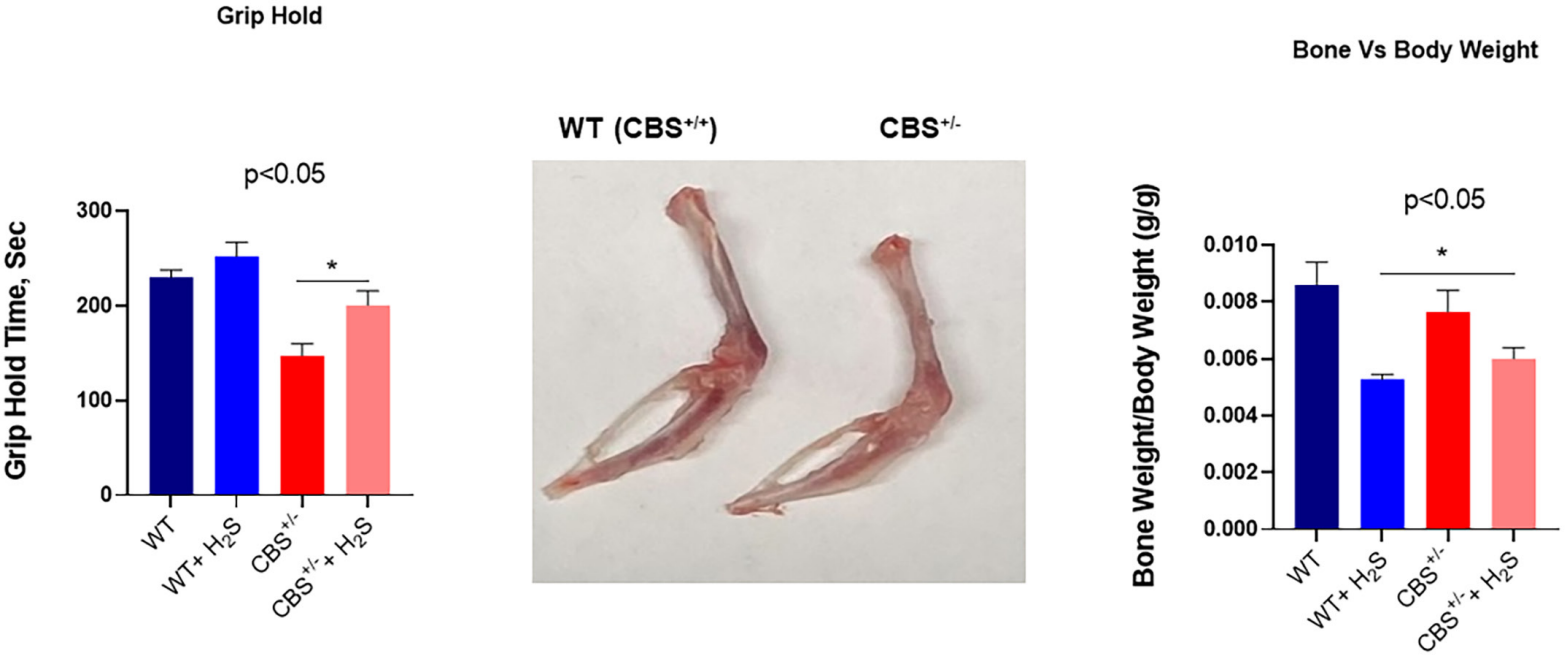
The muscle strength was measured by the grip strength test. The grip strength of the CBS^+/−^ mice was significantly decreased as compared to the WT control. The H_2_S treatment reversed the decrease in the grip strength of CBS^+/−^ mice. Representative tibia, fibula, and the femur bone photographs are shown, *p < 0.05; *n* = 5–12.

Remodeling by its very nature implies the synthesis and degradation of the extracellular matrix (ECM). In that context, we detected an increased level of MMP2 (Figure 4), and collagen fibrosis (Figure 5) in the CBS^+/−^ mice. The H_2_S treatment mitigated these effects too. To further determine whether the increase in fibrosis causes a decrease in skeletal muscle blood flow, we went on to measure the hind‐limb blood flow in the mice. The results showed decreased blood flow in the CBS^+/−^mice. The H_2_S treatment reversed this decrease in the blood flow to almost normal levels (Figure 6). Finally, we decided to measure the strength of the skeletal muscle and bone by the grip test and bone density. The results clearly suggested a decrease in muscle strength in the CBS^+/−^mice and bone strength. Interestingly, the H_2_S treatment ameliorated the decrease in strength in a muscle, and bone in CBS^+/−^ (HHcy) mice (Figure 7).

**FIGURE 8:**
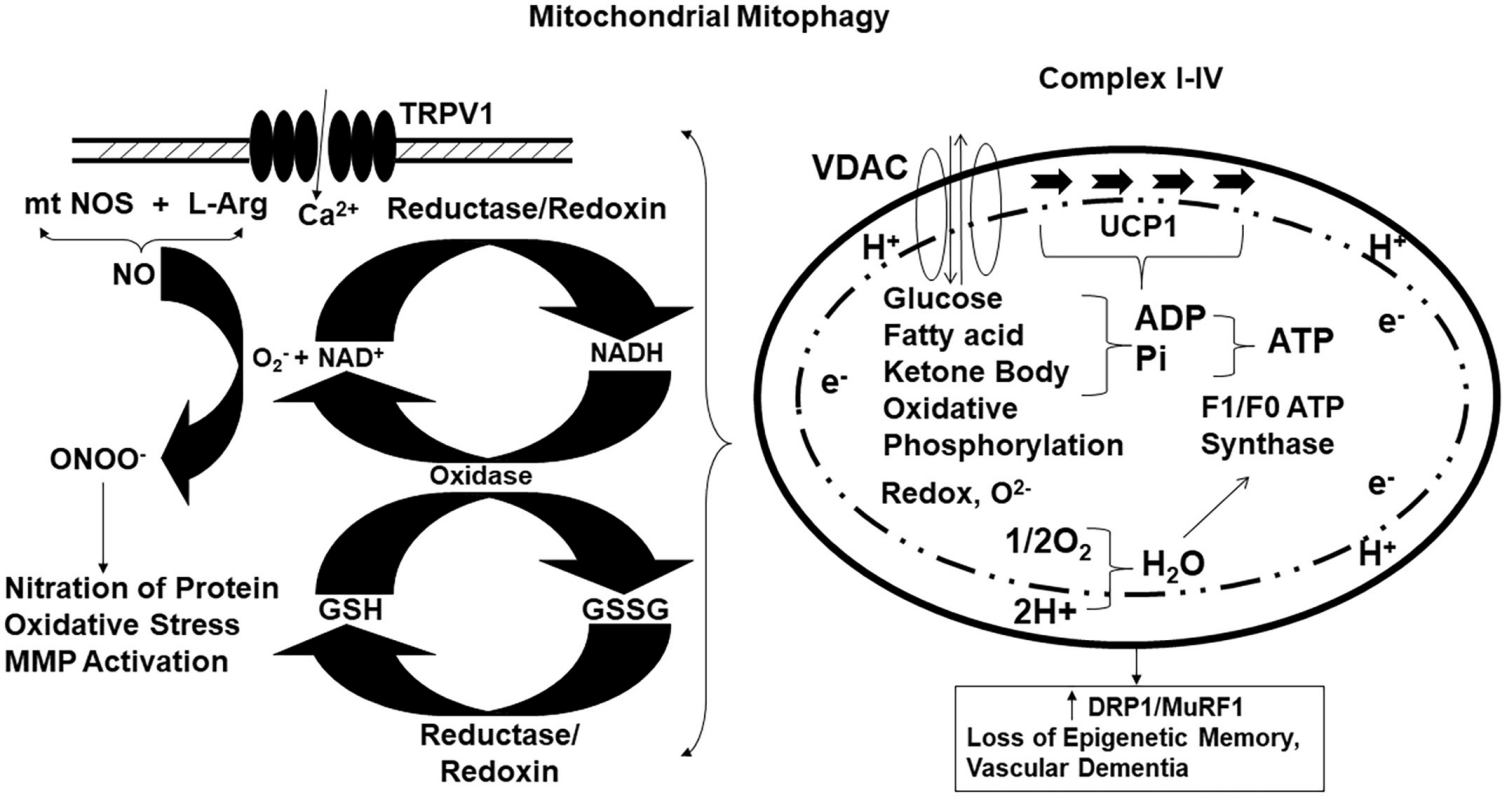
The conclusion supports the mitochondrial mitophagy (i.e., increase in DRP1/MuRF1 ratio) in the CBS^+/−^ mice skeletal muscle. The H_2_S treatment normalized the mitochondrial bioenergetics, successfully.

In conclusion, our data support the hypothesis that an increase in gene writer, and a concomitant decrease in eraser cause mitochondrial fission, and mitophagy that make the skeletal muscle weak in the hyperhomocysteinemic (HHcy) mice (Figure 8). An intervention via the H_2_S treatment did help normalize these musculo‐skeleton abnormal changes.

## 4 DISCUSSION

We have found that HHcy promotes skeletal muscle atrophy, and injury through the epigenetic disruption of the gene writer, and eraser function. The gene writer (DNMT) and eraser (FTO) were altered (Ijiri et al., 2005; Sendžikaitė et al., 2019). HHcy chronically activates MMP‐2 and MMP‐13 associated with GADD45b and TET2 which causes a decrease in epigenetic eraser (Gu et al., 2020). That aspect induces mitochondrial mitophagy (Bayraktar & Kreutz, 2018b; Gavin et al., 2013; Singh et al., 2021; Subbotina et al., 2015; Tyagi et al., 2011). These responses were also elevated in CBS^+/−^mice. Additionally, we found HHcy decreases musclin levels in skeletal muscle tissue, therefore, decreases skeletal muscle physical endurance and impairs mitochondria oxidative capacity. H_2_S treatments mitigate these impairments. Also, HHcy induces lower body mass, poor physical performance, and enhanced atrophic responses. These findings suggest that HHcy is detrimental to skeletal muscle homeostasis and long‐term function. Our study investigates the affected signal mechanisms in three key areas: tissue perfusion, tissue regeneration, and muscle atrophy. Furthermore, it showed the prevention of progressive muscle impairments in HHcy by either NaHS administration or reducing the Hcy levels and enhancing H_2_S levels in skeletal muscle tissue milieu in CBS heterozygous knockout mice.

## AUTHOR CONTRIBUTIONS

MS and SCT designed the research; PK, YZ, RPH, and IS performed the experiments. RPH, MS, and SCT analyzed the data and interpreted the results. MS, SPM, and SCT drafted, edited, and finalized the manuscript. All authors approved the final version of the manuscript before its submission.

## CONFLICT OF INTEREST

No conflicts of interest, financial or otherwise.

## ETHICAL APPROVAL

The experiments in this study were performed in accordance with the relevant guidelines and regulations.
